# Transactions of the Intercolonical Medical Congress of Australasia

**Published:** 1890-06

**Authors:** 


*Transactions of the Intercolonial Medical Congress of Australasia.* Second Session. January, 1889. Melbourne: STILLWELL & CO.

Our Australasian brethren have, in this bulky volume of over 1,000 pages, presented us with a series of papers on Medicine and Surgery which are worthy of being placed on a level with some of the most valued archives of our art. Pre-eminent in value are the papers and discussions on Hydatid Disease: these must in future be regarded as landmarks in our study of the subject. The section on Hygiene also was particularly fruitful of good work. The *Transactions* are worthy of the highest praise, and reflect credit on all concerned.

